# Trends in rheumatoid arthritis burden in China and globally, 1990–2021: A longitudinal study based on the GBD database

**DOI:** 10.1371/journal.pone.0323372

**Published:** 2025-05-21

**Authors:** Zhengpeng Li, Hao Zeng, Bo Jin, Mianyu Zhang, Xiaoyun Zhang, Yuan Chai

**Affiliations:** 1 Guangxi University of Chinese Medicine, Nanning, China; 2 Department of Orthopedics, Ruikang Hospital, Guangxi University of Chinese Medicine, Nanning, China; 3 Suzhou TCM Hospital Affiliated to Nanjing University of Chinese Medicine, Suzhou, China; 4 Nanjing University of Chinese Medicine, Nanjing, China; Shaanxi Provincial People's Hospital, CHINA

## Abstract

**Background:**

Rheumatoid arthritis (RA) is a chronic autoimmune disease that imposes significant health and economic burdens worldwide, particularly in developing countries such as China.

**Method:**

In this study, the Global Burden of Disease, The GBD database system stratified the incidence,prevalence,death and disability-adjusted life years (DALYs) of RA in China and the world from 1990 to 2021 by age, sex and period. Joinpoint Regression Program 5.1.0 was then used to calculate average annual percent change (AAPC) and 95% confidence interval (95% confidence interval). CI) to identify trends in disease burden. In addition, data collation using WPS allows for the comparison of RA burdens across different age groups, genders and time points in China and globally, and the data is rigorously screened and processed to ensure accuracy and comparability.

**Results:**

From 1990 to 2021, ASIR of RA in China increased by 0.54%, while the global ASIR rose by 0.41%.The number of RA cases in China increased cumulatively by 133%, compared to a global increase of 125.21%.Simultaneously, ASPR in China and globally increased by 17% and 14.44%, respectively.Regarding mortality, although the number of RA–related deaths increased in both China and globally, ASMR decreased by 26.23% in China and 22.86% globally.The trend in ASDR was consistent with ASMR, with declines of 0.40% in China and 1.46% globally.Furthermore, the study revealed significant gender disparities in RA both in China and globally, with women experiencing higher incidence, prevalence, mortality, and DALYs than men.The burden of RA increased significantly with age, particularly among middle–aged and older adults aged 45 and above.

**Conclusion:**

Over the past 30 years, the burden of RA in China and globally has undergone significant changes.The study found that while RA related mortality and DALYs have slightly decreased in China and globally, the incidence and prevalence rates have continued to rise, particularly among women and middle–aged to elderly populations.Population aging and changes in lifestyle are key drivers of the increasing RA burden, with women showing higher susceptibility and burden due to their unique physiological characteristics and societal roles.The study highlights the need to strengthen early screening and intervention, optimize personalized treatment plans, and pay special attention to the unique needs of elderly and female populations.Additionally, promoting healthy lifestyles, improving primary healthcare services, and implementing supportive policies can effectively alleviate the health and socioeconomic burden of RA, ensuring a better quality of life for patients.

## Introduction

Rheumatoid arthritis(RA) is a complex and persistent systemic autoimmune disease, characterized by chronic inflammation of the synovial membrane, vasculitis, and progressive bone destruction [1]. This disease tends to cause symmetric inflammatory responses in small joints, with patients often experiencing joint stiffness (morning stiffness) upon waking, accompanied by redness, swelling, and severe pain.In the early stages, these types of discomforts are commonly observed in the proximal interphalangeal joints. Over time, however, the affected areas may gradually extend to other major joints, including the wrists, feet, knees, and even hips [2]. If the progression of RA is not effectively controlled, it will inevitably lead to severe joint dysfunction and even deformity, significantly limiting the patient's ability to perform daily activities [3]. In extreme cases, the disease may invade vital organs such as the heart and lungs, further exacerbating the deterioration of the patient's health, potentially rendering them completely unable to work and incapable of independent living [4]. Recent epidemiological studies have revealed a concerning trend: from 1990 to 2017, the global age–standardized incidence rate of RA increased significantly by 8.2%.In China, this severe health challenge is equally prominent, with the number of RA patients exceeding 5 million. The male–to–female prevalence ratio is as high as 1:4, indicating that women are more vulnerable to RA and bear a disproportionately heavier disease burden compared to men [5,6]. RA not only poses a severe threat to the physical and mental health of individual patients but also imposes a substantial economic burden on their families and the broader socioeconomic system.The high cost of treatment, long–term care requirements, and reduced income due to limited work capacity collectively create a complex and extensive economic burden [7,8]. Consequently, RA has emerged as a pressing public health issue that demands heightened attention from all sectors of society and the implementation of effective measures.To address this challenge, we must increase research funding, raise public awareness, and optimize diagnostic and treatment strategies to offer patients a brighter future.

With the intensification of population aging, shifts in lifestyle, widening cognitive disparities, and rapid advancements in healthcare, the incidence trends and disease burden of RA in China and globally are expected to exhibit significant regional differences [9,10]. Despite the rapid advancements in global medical technology over recent decades, which have significantly reduced disability rates and alleviated disease burdens, the overall burden of RA remains severe and cannot be overlooked due to the uneven distribution of medical resources worldwide and relatively weak public health awareness [11]. As the world's largest developing country, China has made significant progress in the medical field, yet there remains a gap in healthcare standards compared to developed countries.Notably, the lack of public awareness and emphasis on early prevention and standardized treatment of RA presents numerous challenges and difficulties for its diagnosis and treatment in China [12]. Therefore, closely monitoring and tracking the temporal trends in the burden of RA has become an essential prerequisite for formulating effective public health strategies.

The Global Burden of Disease (GBD) database is an indispensable tool for evaluating disease trends globally, as well as at national and regional levels. It provides us with comprehensive and reliable epidemiological data and analytical methods [13]. Leveraging the valuable resource of the GBD database, we can access a diverse set of metrics for measuring disease burden, encompassing dimensions such as incidence, prevalence, mortality, and DALYs. This enables comprehensive and in–depth assessments of disease burden trends at global and country–specific or regional levels, providing policymakers in the public health sector with invaluable perspectives and data support [14]. Building on this foundation, this study leverages the robust capabilities of the GBD database to meticulously extract detailed data on RA in China and globally from 1990 to 2021, including but not limited to incidence, prevalence, mortality, DALYs, as well as key demographic information such as gender and age distribution.Through a series of systematic and in–depth analyses, this study aims to uncover significant differences between China and the global context in terms of RA across temporal, age, and gender dimensions, while exploring its developmental trends and evolution.These findings not only contribute to a more comprehensive and profound understanding of the intrinsic characteristics of this complex disease but also provide a solid foundation and guidance for formulating evidence–based public health policies and implementing effective RA control measures.By accurately capturing the trends of RA, future efforts can more effectively allocate medical resources and optimize prevention strategies, ultimately reducing the global burden of RA and improving patients’ quality of life and well–being.

## Data and methods

### Data sources

The data for this study were sourced from the GBD 2021 database, meticulously developed by the renowned Institute for Health Metrics and Evaluation (IHME) at the University of Washington.IHME employs a series of standardized and highly comparable methods to conduct comprehensive, systematic, and scientific evaluations of the burden of over 300 diseases and injuries across 204 countries and territories worldwide.In this process, the center also incorporates more than 80 risk factors closely related to disease burden, thoroughly integrating and meticulously organizing their attributed impacts on disease burden, thereby ensuring data comprehensiveness and accuracy [13]. In this study, we employed two standardized tools widely used in the field of global disease burden research—Disability Model–Mixed Effects Regression DisMod–MR2.1 and the Cause of Death Ensemble Model (CODEm) to perform estimations.DisMod–MR2.1, an advanced Bayesian meta–regression disease modeling tool, excels in integrating fragmented and heterogeneous epidemiological data on non–fatal diseases and injuries. By comprehensively evaluating data on incidence, prevalence, mortality, and age–standardized DALYs, it significantly enhances the consistency among epidemiological parameters, ensuring precision and reliability in data estimation [15]. CODEm is a highly integrated analytical tool designed for in–depth analysis of cause–of–death data. It seamlessly integrates a variety of modeling strategies to accurately analyze ratios or cause fractions and rigorously tests out–of–sample predictive validity, carefully selecting the most optimal covariates to ensure the accuracy and reliability of the analysis results [16,17]. This study utilized the online query platform (http://ghdx. healthdata.org/gbd-results-tool/) to meticulously select and process GBD 2021 data. During the query, we set the location to “China, Global,” the cause to “Rheumatoid Arthritis,” and the time span to “1990–2021,” with age groups divided into 5–year intervals, ranging from “15–19 years” to “ ≥ 95 years.” Subsequently, we precisely extracted authoritative data on the incidence, mortality, prevalence, and DALYs of RA from 1990 to 2021 from the GBD database. Given the public availability of the GBD 2021 data, this study was exempted from requiring additional approval from the institutional ethics committee. Throughout the research process, we adhered strictly to the Guidelines for Accurate and Transparent Health Assessment Reporting to ensure the study's scientific rigor and credibility.

### Statistical analysis

This study utilized GBD 2021 data to focus on the incidence, prevalence, mortality, DALYs, and corresponding annual standardized metrics for RA in China and globally, including age–standardized incidence rate(ASIR), age–standardized prevalence rate(ASPR), age–standardized rates of mortality(ASMR), and age–standardized DALY rate(ASDR). Additionally, crude rates for incidence (CIR), prevalence (CPR), mortality (CMR), and DALYs across different age groups were used to comprehensively depict the disease burden of RA and its trends in China and globally. Furthermore, all standardized rates were calculated based on the age structure of the GBD world standard population to eliminate the influence of age composition differences, ensuring a more scientific and accurate assessment of RA–related disease burden across different period.

### Statistical evaluation

This study utilized WPS to systematically organize RA–related data. Following the age grouping standards published in GBD 2021, we selected individuals aged 15 years and above as the study population, dividing them into 14 age groups with 5–year intervals. On this basis, we conducted a comparative analysis of the distribution characteristics of ASIR, ASPR, ASMR, and ASDR among RA patients across different age groups and genders in China and globally from 1990 to 2021. Additionally, using the Joinpoint Regression Program 5.1.0 software developed by the U.S. National Cancer Institute, we investigated the trends in standardized rates of RA burden indicators in China and globally from 1990 to 2021. Using a log–linear regression model, we precisely calculated the average annual percent change (AAPC) and its 95% confidence intervals (CIs) [18]. If the lower limit of the AAPC 95% CI exceeds 0, it indicates an upward trend in the metric. If the upper limit of the AAPC 95% CI is below 0, it indicates a downward trend. If the 95% CI range includes 0, the trend change is not statistically significant. The significance level was set at α = 0.05 [19,20]. Additionally, this study utilized R statistical software (version 4.3.3) for statistical analysis and data visualization.

### Ethical considerations

As this study used publicly available, de-identified data from the GBD 2021, no ethical approval was necessary. All procedures adhered to applicable ethical standards and regulations.

## Result

### The incidence of RA in China and the world

The incidence number of RA in China increased from 127,826(95% CI:111,477–145,914) in 1990–247,307(95% CI: 216,205–282,998) in 2021, representing a cumulative increase of 93.47%. Globally, the incidence number of RA rose from 488,269 (95% CI: 435,015–545,895) to 1,000,319 (95% CI: 902,687–1,114,213), reflecting a cumulative increase of 104.87%. The ASIR of RA in China increased from 11.59 per 100,000 population (95% CI:10.15–13.15) in 1990 to 13.70 per 100,000 (95% CI: 12.12–15.55) in 2021. Globally, the ASIR of RA increased from 10.43 per 100,000 population (95% CI: 9.32–11.64) in 1990 to 11.80 per 100,000 (95% CI: 10.64–13.12) in 2021. Meanwhile, the AAPC of RA incidence increased by 0.54% in China and 0.41% globally (Table 1).

**Table 1:** All–age cases and age–standardised incidence, prevalence, mortality, DALYs and corresponding AAPC for RA in China and world in 1990 and 2021.

Location	Measure	1990		2021		1990–2021 AAPC
		**All–Ages cases**	**Age–standardizedrates per 100,000 people**	**All–Ages cases**	**Age–standardizedrates per 100,000 people**	
		**n(95% CI)**	**n(95% CI)**	**n(95% CI)**	**n(95% CI)**	**n(95% CI)**
**China**						
	Incidence	127,826 **(**111,477–145,914**)**	11.59 **(**10.15–13.15**)**	247,307 **(**216,205–282,998**)**	13.70 **(**12.12–15.55**)**	0.54 **(**0.53–0.56**)**
	Prevalence	2041,682 **(**1,746,913–2,391,022**)**	205.70**(**177.56–238.18**)**	4,755,487 **(**4,141,219–5,452,492**)**	240.70**(**210.77–277.95**)**	0.51 **(**0.49–0.53**)**
	Deaths	4,776 **(**3,903–5,934**)**	0.70 **(**0.57–0.85**)**	10,279 **(**7,411–12,615**)**	0.54 **(**0.39–0.66**)**	–0.78 **(**–1.20 – –0.37**)**
	DALYs	403,058 **(**307,641–526,244**)**	42.37 **(**33.04–54.32**)**	833,818 **(**621,520–1,083,523**)**	42.20 **(**31.30–55.45**)**	–0.02 **(**–0.13–0.09**)**
**Worldwide**						
	Incidence	488,269 **(**435,015–545,895**)**	10.43 **(**9.32–11.64**)**	1,000,319 **(**902,687–1,114,213**)**	11.80 **(**10.64–13.12**)**	0.41 **(**0.39–0.42**)**
	Prevalence	7,959,055 **(**7,041,419–9,085,469**)**	182.54 **(**161.59–207.48**)**	17,924,667 **(**1,597,3178–20,303,303**)**	208.90**(**186.34–236.33**)**	0.44 **(**0.42–0.46**)**
	Deaths	21,671 **(**19,287–24,202**)**	0.61 **(**0.54–0.68**)**	37,330 **(**31,060–43,136**)**	0.45 **(**0.37–0.52**)**	–0.95 **(**–1.08 – –0.81**)**
	DALYs	1,545,699 **(**1,201,479–1,977,786**)**	36.42 **(**28.71–45.70**)**	3,075,303 **(**2,310,381–3,974,046**)**	35.89 **(**26.95–46.46**)**	–0.05 **(**–0.10–0.01**)**

### The prevalence of RA in China and the world

In China, the prevalence number of RA increased from 2,041,682(95% CI:1,746,913–2,391,022) in 1990–4,755,487(95% CI: 4,141,219–5,452,492) in 2021, representing a cumulative increase of 133%. Globally, the number prevalence of RA cases rose from 7,959,055(95% CI:7,041,419–9,085,469) in 1990–17,924,667(95% CI:15,973,178–20,303,303) in 2021, reflecting a cumulative increase of 125.21%. On the other hand, the ASPR of RA in China increased from 205.70 per 100,000 population(95% CI: 177.56–238.18) in 1990 to 240.70 per 100,000(95% CI:210.77–277.95) in 2021, representing a 17% increase. Globally, the ASPR of RA increased from 182.54 per 100,000 population (95% CI:161.59–207.48) in 1990 to 208.90 per 100,000 (95% CI:186.34–236.33) in 2021, reflecting a 14.44% increase. Meanwhile, from 1990 to 2021, the AAPC of RA prevalence increased by 0.51% in China and by 0.44% globally (Table 1).

### The mortality of RA in China and the world

In 2021, the number of mortality caused by RA in China reached 10,279(95% CI: 7,411–12,615), a sharp increase of 115.05% compared to 1990. Globally, the number of RA–related mortality rose by 72.27% in 2021 compared to 1990. However, from 1990 to 2021, the ASMR of RA showed a declining trend both globally and in China. In China, the ASMR of RA decreased from 0.70 per 100,000 population to 0.54, a reduction of 22.86%. Globally, the ASMR declined from 0.61 per 100,000 population to 0.45, representing a reduction of 26.23%. Additionally, from 1990 to 2021, the AAPC of global RA mortality declined by 0.95%, while in China, it declined by 0.78% (Table 1).

### The DALYs in China and the world

In China, the number of DALYs due to RA increased from 403,058 (95% CI:307,641–526,244) in 1990–833,818 (95% CI:621,520–1,083,523) in 2021, representing a growth of 106.88%. During the same period, global DALYs increased by 99.02%.Regarding ASDR, in China, it decreased from 42.37 per 100,000 population (95% CI:33.04–54.32) in 1990 to 42.20 per 100,000 (95% CI:31.30–55.45) in 2021, a reduction of 0.40%. Globally, the ASDR decreased from 36.42 per 100,000 population(95% CI:28.71–45.70) in 1990 to 35.89 per 100,000 (95% CI:26.95–46.46) in 2021, a reduction of 1.46%. Additionally, from 1990 to 2021, the AAPC of global DALYs declined by 0.05%, while in China, the AAPC declined by 0.02% (Table 1).

### Joinpoint regression analysis of RA burden in China and the world

**Fig 1:**
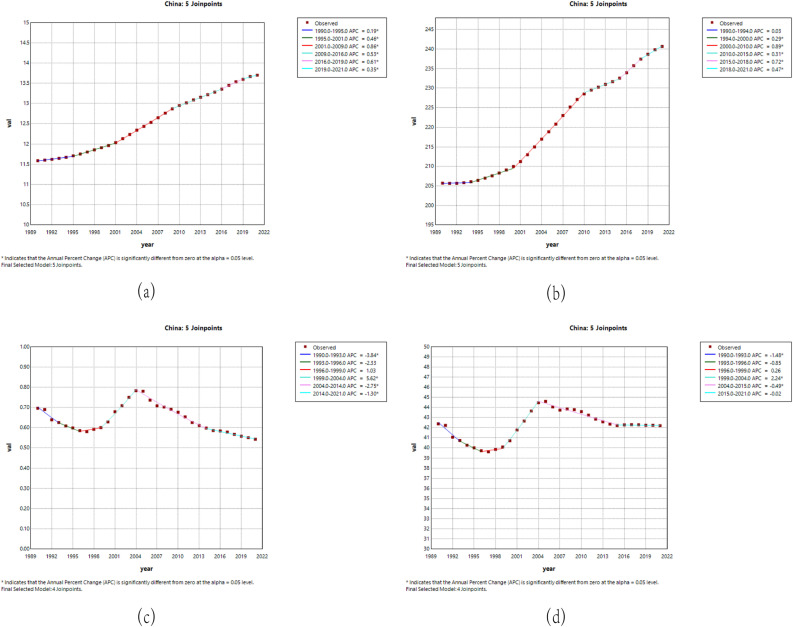
APC of ASIR, ASPR, ASMR and ASDR for China RA from 1990 to 2021. (a) ASIR;(b) ASPR;(C) ASMR;(d) ASDR.

Joinpoint regression analysis revealed trends in ASIR, ASPR, ASMR, and DALYs for RA burden in China and globally from 1990 to 2021. The analysis indicated that during 1990–2021, the annual percentage change(APC) for ASIR and ASPR in China significantly increased (P < 0.05). Specifically, ASMR in China showed an upward trend from 1999 to 2004(APC = 5.62, P < 0.05), followed by a downward trend from 2004 to 2021 (P < 0.05). Overall, the ASMR in China exhibited a declining trend. Regarding ASDR in China, it showed an upward trend from 1999 to 2004 (P < 0.05). After 2004, despite fluctuations, the ASDR tended to stabilize, with levels slightly higher than before.In contrast, ASPR globally has shown an upward trend since 1994 (P < 0.05). ASMR and ASDR exhibited a downward trend from 1990 to 1998 (P < 0.05), followed by an upward trend from 1998 to 2003 (P > 0.05). Globally, ASIR trends were similar to those in China, showing an upward trend (P < 0.05) (Figs 1 **and** S1 Fig).

### The trends in China and globally of RA disease burden

**Fig 2:**
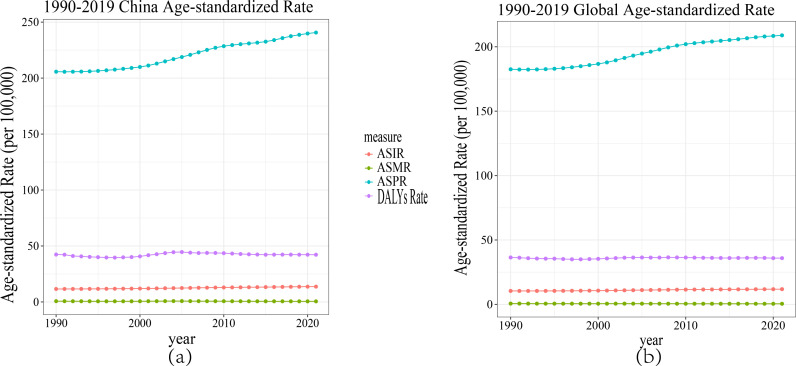
Comparison of ASIR, ASPR, ASMR and ASDR trends in China and global RA from 1990 to 2021. (a) China; (b) Global.

Analysis of trends from 1990 to 2021 reveals distinct characteristics in the RA burden in China and globally. In China, the ASPR of RA has been continuously increasing, with a growth rate significantly exceeding the global average. Meanwhile, the global ASPR is also increasing but at a slower rate and remains lower than that of China, indicating a relatively moderate growth in the global RA burden. The ASIR of RA in China has slightly increased, with a limited growth rate similar to the global trend. Moreover, the ASMR and ASDR of RA in both China and globally have remained relatively stable, suggesting that despite an increase in prevalence, the impact of RA on life expectancy has not significantly worsened. This may be attributed to advancements in medical technology and improved disease management strategies. Overall, the RA burden in China has increased more significantly, whereas globally, it shows a relatively steady growth trend (Fig 2).

### Analysis of RA burden in different age groups in China and the world in 1990 and 2021

**Fig 3:**
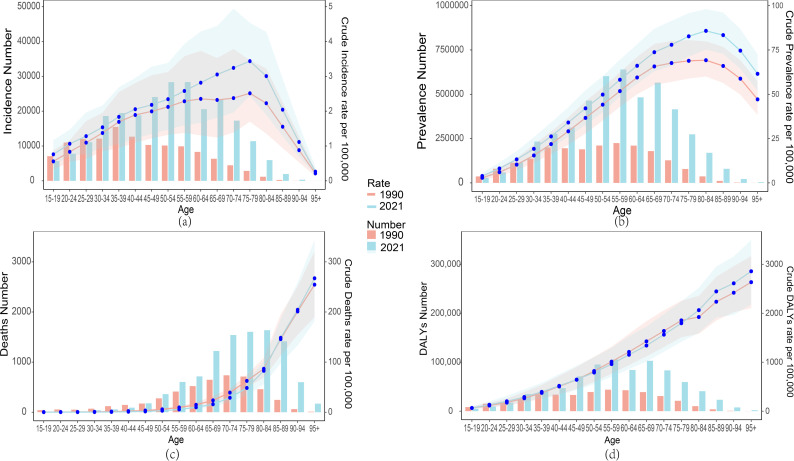
Comparison of the number of incidence, prevalence, deaths and DALYs counts by age group and their crude ratios in China in 1990 and 2021. (a) Number of incidence and CIR; (b) Number of prevalence and CPR; (c) Number of deaths and CMR; (d) Number of DALYs and CDR.

A comparison of the incidence, prevalence, mortality, and DALYs of RA across different age groups in China between 1990 and 2021, along with the corresponding crude rates, reveals the following trends. Regarding incidence and prevalence, in 1990, the highest number of RA cases in China and globally were concentrated in the 35–39 age group (Figs 3a **and** S2 Fig). This age group also had the highest prevalence in China (Fig 3b), whereas globally, the highest prevalence was in the 60–64 age group (S2 Fig). By 2021, the highest number of RA cases in China and globally were concentrated in the 50–54 and 54–59 age groups (Figs 3a**、**b **and** S2 Fig).In China, the CIR showed a fluctuating upward trend between the 15–19 and 75–79 age groups in both 1990 and 2021, with a sharp decline observed in those aged 80 and above (Fig 3a). Globally, the CIR exhibited a fluctuating upward trend from the 15–19 to the 65–69 age groups, with a gradual decline after 70 years of age (S2 Fig). Additionally, the peak CPR in China occurred in the 80–84 age group, while globally, it was in the 70–75 age group. However, both trends followed a similar upward pattern (Figs 3b **and** S2 Fig).

With respect to mortality, in 1990, the peak number of RA–related deaths in China was in the 70–74 age group, while in 2021, it shifted to the 80–84 age group (Fig 3c). Globally, the peak number of RA–related deaths in both 1990 and 2021 was in the 75–79 age group (S2 Fig). Both in China and globally, the CMR increased with age from 1990 to 2021, showing an upward trend. A similar trend was observed in the CDR, with an increase as age advanced.In 1990 and 2021, the peak DALYs in China occurred in the 55–59 age group, whereas globally, it occurred in the 65–69 age group (S2 Fig). These two Fig illustrate significant changes in the number of cases, prevalence, mortality, and DALYs for RA across different age groups in China and globally in 2019 and 2021.

Overall, both in China and globally, the disease burden significantly increases with age, particularly among middle–aged and elderly groups over 45, where incidence and prevalence rapidly rise, peaking between 60 and 74 years of age.Global data shows trends similar to China, with incidence and prevalence increasing significantly after 45, peaking between 60 and 75, and then gradually declining. Particularly in the elderly group above 80, mortality and DALYs remain at higher levels, reflecting the highest health risks in this age group.Compared to 1990, the disease burden in 2021 significantly increased, with a particularly notable rise in prevalence and DALYs (Figs 3 **and** S2 Fig).Both in China and globally, the disease burden remains relatively stable in young and middle–aged adults, then increases rapidly after 45, peaking in the elderly group, and gradually declines after 90 years of age (Figs 3 **and** S2 Fig).

### Gender differences in RA burden in China and the world

**Fig 4:**
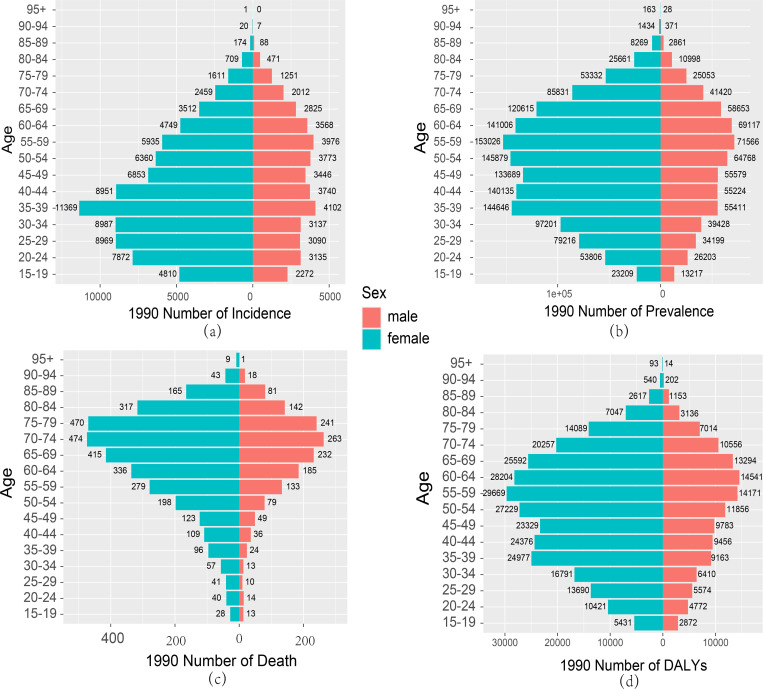
Comparison of RA incidence, prevalence, death and DALYs among men and women of different age groups in China in 1990. (a) Incidence;(b)Prevalence;(c) Deaths;(d) DALYs.

**Fig 5:**
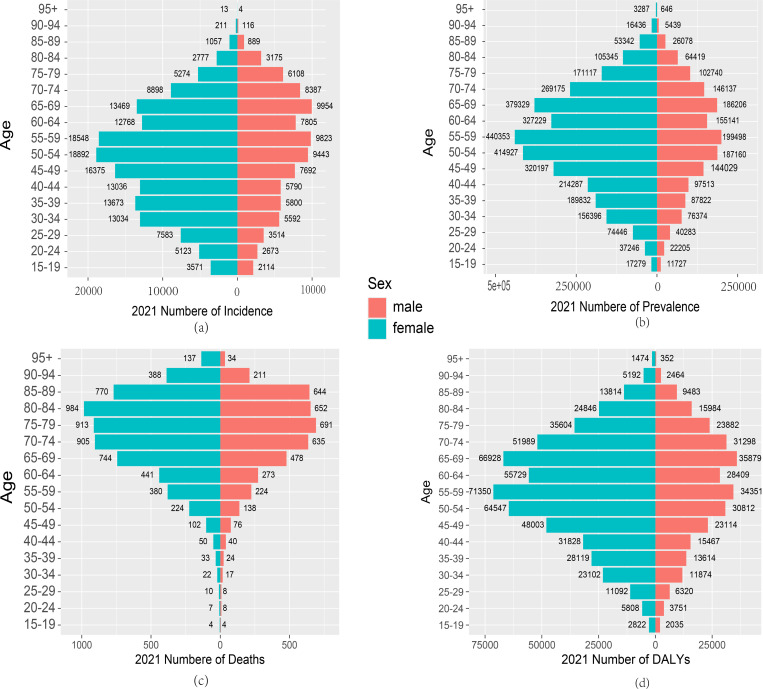
Comparison of RA incidence, prevalence, death and DALYs among men and women of different age groups in China in 2021. (a) Incidence;(b)Prevalence;(c) Deaths;(d) DALYs.

Research present the comparative results of RA incidence, prevalence, mortality, and DALYs for different age groups of males and females in China and globally in 1990 and 2021. Regarding incidence, in 1990, the number of male RA cases in China was lower than that of females (Fig 4a), and globally, males had fewer cases than females in all age groups except for the 15–19 age group (S3 Fig). In 1990, both male and female RA incidence peaked in the 35–39 age group (Fig 4a), while in 2021, the peak incidence shifted to the 50–54 age group for both sexes, with males reaching the peak at 65–69 years (Fig 5a).Regarding prevalence, in 1990, the number of cases increased progressively from the 35–64 age group, peaking particularly in the 55–59 age group (Fig 4b). By 2021, prevalence increased across all age groups, particularly between the 40–64 age groups (Fig 5b). Additionally, the 2021 data shows a significant increase in prevalence among those aged 65 and above, particularly between the 65–84 age groups, where prevalence reached a new peak (Fig 5b).

From a gender perspective, the number of female cases in 1990 and 2021 was higher than that of males, and this trend was consistent globally (Figs 4,5 **and** S3,4). Additionally, compared to 1990, the number of male and female cases decreased in several age groups in 2021, with females in the 15–29 age range across three groups, and males in the 15–24 age range across two groups (Figs 4b **and** Fig 5b).Compared with that in 1990, the number of deaths increased in most age groups in 2021, especially in the elderly group aged 85 and above (Figs 4c **and** Fig 5c). In 2021,whether in China or global there was a significant increase in the number of deaths in the elderly population aged 60 and above (Figs 4c **and** S4 Fig). Additionally, in 1990, female mortality was higher than male mortality across all age groups, and in 2021, female mortality remained higher (Figs 4c **and** Fig 5c). Moreover, male mortality in the 80 and above age group also significantly increased (Figs 4c **and** Fig 5c). In contrast, globally, the number of female deaths in 1990 and 2021 was much higher than that of males in all age groups (S3 **and** 4 Figs).

The DALYs results in China mirrored the prevalence and mortality trends. In both 1990 and 2021, female DALYs were higher than those of males across all age groups (Fig 4d **and** Fig 5d). In 1990, the peak DALYs for females occurred in the 55–59 age group, while for males, it occurred in the 60–64 age group (Fig 4d). In 2021, the peak DALYs for both females and males occurred in the 55–59 age group (Fig 5d). In contrast, the global DALYs results in 1990 and 2021 were consistent with the global mortality trends (S3 **and** 4 Figs ).

**Fig 6:**
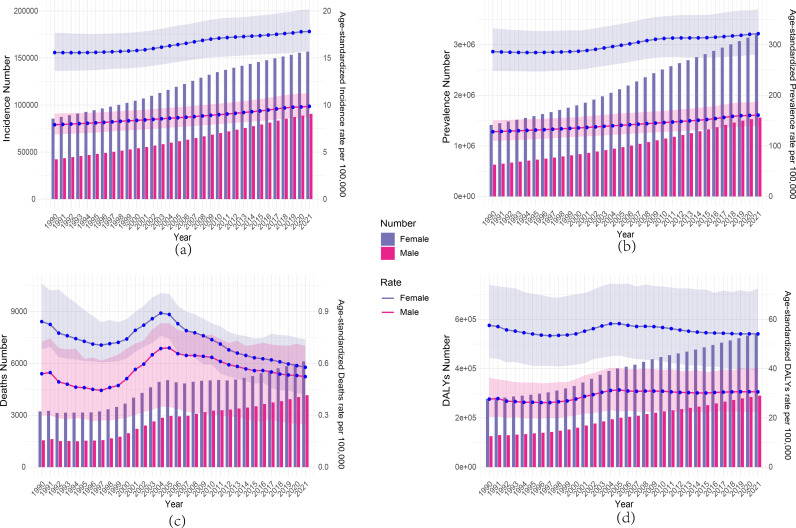
Comparison of all–age cases with age–standardized incidence, prevalence,deaths, and DALYs in Chinese men and women, 1990–2021. (a) Number of incidence and ASIR; (b) Number of prevalence and ASPR; (c) Number of deaths and ASMR; (d)Number of DALYs and ASMR.

Our study also show the comparison of RA burden and age–standardized rates for males and females in China and globally from 1990 to 2021. Fig 6a shows the ASIR for males and females in China and globally. In China, the ASIR increased consistently throughout the period, with females always higher than males. However, globally, the ASIR stabilized after 2000, and the gap between males and females decreased, with females still showing a slightly higher ASIR than males (S5 Fig). In Fig 6b, the ASPR for both males and females in China increased steadily over time, following a trend similar to ASIR. In contrast, globally, the ASPR fluctuated between 1990 and 2005, then leveled off (S5 Fig). Additionally, the ASPR for females was significantly higher than for males in both China and globally, and the gap gradually decreased over time (Figs 6b **and** S5 Fig).

Furthermore, Fig 6 and S5 display the ASMR trends for males and females in China and globally. In both China and globally, the ASMR for females was higher than for males, with both showing an overall downward trend. The ASMR in China fluctuated considerably, with an increase from 1990 to 1991, a decline from 1991 to 1997, an increase from 1997 to 2005, and a gradual decline after 2005 (Fig 6c). In contrast, the global ASMR showed a relatively smooth decline, with a smaller gap between males and females, particularly after 2010, when the ASMR stabilized (S5 Fig). Fig 6d and 11d show the DALYs situation in China and globally. In China, DALYs for both males and females increased between 2000–2005, but while male DALYs gradually declined over time, female DALYs showed a fluctuating upward trend (Fig 6d). Globally, DALYs for both males and females increased year by year, but the growth rate was slow (S5 Fig).

## Conclusion

This study systematically evaluated the trends in RA burden in China and globally from 1990 to 2021, providing a comprehensive comparison of incidence, prevalence, mortality, and DALYs to highlight differences and trends across time, gender, and age groups. The results indicated that both China and global ASIR and ASPR showed a continuous upward trend with significant growth, while China’s ASMR and ASDR exhibited considerable fluctuations but an overall declining trend. In contrast, global ASMR and ASDR remained relatively stable or showed a slight decline. Additionally, RA prevalence, incidence, mortality, and DALYs, as well as their age–standardized rates, were consistently higher in females compared to males. From an age perspective, RA cases and incidence were predominantly concentrated in middle–aged and elderly populations, with higher mortality rates observed among older adults. These findings suggest that RA prevention and management remain a significant challenge both in China and globally.

From 1990 to 2021, the growth rate of RA prevalence in China was significantly higher than the global level. During this period, China's ASPR increased by approximately 17%, compared to a global increase of about 14.44%. In terms of incidence, China's ASIR grew by 18.2%, while the global ASIR increased by 13.14%. This trend aligns with findings from a global RA burden analysis [21]. This difference may be attributed to changes in population structure, accelerated aging, and increased public awareness of RA. As the most populous developing country in the world, China faces a rapidly aging population, which may partially explain the faster increase in RA prevalence [22]. Moreover, modern lifestyles, including sedentary behavior, dietary changes, and reduced physical activity, have also increased the risk of developing RA. Particularly, with the accelerated urbanization in China, a large rural population has migrated to cities, and the resulting new lifestyles have, to some extent, impacted health and contributed to the growing burden of chronic diseases [6,23]. Additionally, with the improvement of public health awareness, more individuals are actively seeking early diagnosis and treatment, which may have led to the identification of more RA cases, thereby contributing to the observed increase in incidence rates [2]. Over the past three decades, advancements in RA treatment have significantly reduced the risk of mortality and long–term disability among patients. The widespread use of novel antirheumatic drugs and immunosuppressants, including traditional medications and biologics, has effectively reduced joint inflammation and damage, enabling early disease control, slowing disease progression, and subsequently lowering RA–related mortality and long–term disability [24,25], which is consistent with the findings of this study.The results indicate that although the number of RA–related deaths has increased in China and globally, the age–standardized ASMR has shown an overall declining trend. From 1990 to 2021, the ASMR and ASDR in China and globally decreased by 22.8% and 26.23%, respectively, while the reductions in ASDR were more modest, with decreases of 0.4% and 1.46%, respectively. Meanwhile, this trend can be attributed to the emphasis on public health and the widespread dissemination of health knowledge in both China and globally. The improvement in public health awareness has encouraged individuals to prioritize early disease screening and timely medical consultation, thereby enhancing early diagnosis and treatment coverage and ultimately reducing RA–related mortality and disability rates [26]. Therefore, the author believes that the increase in RA prevalence in China and globally is primarily driven by population aging, changes in lifestyle, and improved health awareness. In the future, efforts should be made to further strengthen early screening and diagnosis of RA, particularly in communities and rural areas with relatively limited medical resources, to ensure that more patients receive timely diagnosis and treatment during the early stages of the disease. Additionally, education on promoting healthy lifestyles should be intensified, guiding residents toward healthier habits, including a balanced diet, increased physical activity, and reduced sedentary behavior. Furthermore, governments and healthcare institutions need to enhance the promotion and accessibility of new medications, ensuring that patients can afford and access effective treatments. These interventions are expected to further reduce RA–related mortality and disability rates, thereby alleviating the social and economic burden of the disease.However, a more detailed such as genetic, hormonal, environmental, population aging, lifestyle, and health awareness factors to the patient burden of RA should be conducted in-depth research.This understanding will enable the development of more targeted and effective strategies for prevention and management, addressing both individual and public health needs. In the future the complexity of these interactions necessitates a robust research agenda, leveraging advanced epidemiological methods and interdisciplinary collaboration to translate findings into clinical practice.

This study demonstrates that the incidence, prevalence, mortality, and DALYs of RA significantly increase with age, showing distinct age–related characteristics. In 1990, the age group with the highest incidence and prevalence of RA in China was 35–39 years old; by 2021, this peak shifted to the 50–54 and 54–59 age groups. This trend shift reflects the rapid socioeconomic development, continuous medical advancements, and increased life expectancy in China since the 1990s. However, with increased economic pressures and work burdens, younger generations have lower fertility rates, leading to a rising proportion of elderly individuals and a growing concentration of chronic disease burdens, such as RA, in middle–aged and elderly populations [27]. Meanwhile, the immune system function in middle–aged and elderly individuals gradually declines, accompanied by hormonal changes and the accumulation of environmental factors, reducing their resistance to chronic diseases and increasing the risk of RA incidence and prevalence [28–31]. In contrast, the global peak of RA incidence and prevalence was concentrated in the 60–64 age group in 1990, but by 2021, it had shifted to the 50–59 age group. This difference may be attributed to variations in healthcare conditions, lifestyles, and the pace of population aging across different countries and regions. Developed countries, with more abundant medical resources and wider availability of early RA screening and treatment measures, may enable earlier diagnosis, resulting in a younger age of onset.RA can cause joint dysfunction and pain, limiting many patients’mobility and their ability to perform basic activities such as walking, dressing, and household chores, thereby significantly reducing their quality of life. Moreover, the progression of RA is closely associated with high mortality rates and DALYs. Furthermore, RA significantly increases the risk of cardiovascular complications [32]. The systemic inflammatory response triggered by RA may impair normal cardiovascular function, leading to endothelial damage and lipid metabolism abnormalities, which in turn raise the likelihood of cardiovascular events such as heart attacks and strokes, further exacerbating the health burden on patients [33,34]. In summary, with the intensification of global aging, the burden of RA is rising both in China and globally. This highlights the need for increased attention to the health of aging populations. Future efforts should focus on improving disease management for this demographic by optimizing personalized treatment strategies and developing comprehensive management plans tailored to the multimorbidity characteristics of elderly patients, including interventions such as pharmacological therapy, physical therapy, and psychological support.Simultaneously, efforts should be made to strengthen the prevention and management of RA–related complications, particularly the monitoring and intervention of cardiovascular disease risks. Healthcare resources should be directed toward aging populations, with a focus on improving the capacity of primary healthcare services to ensure timely diagnosis and treatment for elderly patients. Furthermore, through policy support and health education, societal awareness of elderly RA patients should be enhanced to provide them with more comprehensive protection and support systems.Between 1990 and 2021, there were significant gender differences in the burden of RA in both China and globally. In both China and globally, females exhibited significantly higher incidence, prevalence, mortality, and DALYs of RA compared to males. Substantial evidence also suggests that gender plays a critical role in the onset and progression of RA [35,36]. The underlying reasons for this phenomenon may be related to women's unique physiological characteristics, hormonal fluctuations, and social roles [37]. Studies have shown that women's unique immune system characteristics may make them more susceptible to autoimmune diseases [38]. For example, during special periods such as pregnancy, menopause, and menstruation, hormonal fluctuations may disrupt the balance of the immune system, potentially increasing the risk of developing RA [39,40]. The daily living environment is also an important factor contributing to the risk of RA in women. For instance, women may take on more household chores and caregiving responsibilities in daily life, and the prolonged overuse of hand and foot joints, combined with psychological stress, may contribute to the onset of RA [41,42]. The differences in RA burden across age groups are particularly pronounced in middle–aged and elderly populations. Specifically, DALYs for females peaked in the 55–59 age group, highlighting the prominent role of postmenopausal women in the RA burden. In contrast, postmenopausal women face more chronic health issues, making the impact of RA on their quality of life and overall health more severe [43,44]. These findings suggest that RA prevention and control strategies should take gender differences into account, with a particular focus on physiologically susceptible age groups in women to enhance prevention and early intervention. Regular monitoring, pharmacological interventions, and optimizing work and living environments for women at risk of immune imbalance due to estrogen decline during menopause and postmenopause can help reduce their susceptibility to RA.

This study systematically evaluated the burden of RA in China and globally from 1990 to 2021, highlighting that despite significant advancements in RA treatment technologies, the absence of a cure underscores the importance of early prevention and lifestyle adjustments. The findings suggest that improving diet, increasing physical activity, and adopting other healthy lifestyle interventions can effectively reduce the incidence and progression of RA, particularly among middle–aged and elderly women [45]. Furthermore, strengthening public health strategies and disease education to raise public awareness of the importance of RA prevention is key to reducing the burden of RA. However, this study has certain limitations. Firstly, the data analyzed in this study is current only up to 2021. Although this represents the latest available database at the time of analysis, it has inherent limitations in predicting and analyzing potential new trends and changes that may arise in the future. As a result, there are constraints regarding the timeliness of the data for forward - looking research. Secondly, the study relies on secondary data from the GBD database, although provides extensive data, its multiple sources may introduce errors or omissions during the data collection and integration process, potentially affecting the accuracy of the results. Moreover, it may not accurately reflect the specific conditions of individual regions. Variations in medical resource distribution, environmental factors, and lifestyle habits across different regions could lead to discrepancies between the actual RA burden and the database data, limiting the applicability of the study's conclusions to specific locales.Thirdly, the lack of primary patient-level data limits the ability to perform detailed analyses on individual risk factors or to adjust for all possible confounders.Fourthly, the GBD database does not provide detailed information on genetic, hormonal, or environmental factors, which are important for understanding the etiology and progression of RA. Therefore, future research should aim to investigate these factors to better understand the observed trends.In conclusion, RA is a global public health challenge that significantly impacts individuals, families, and societal health. With the intensification of aging populations globally, particularly in China, the burden of RA is expected to continue increasing. Therefore, implementing effective public health strategies, enhancing public health education, and improving lifestyles will be crucial in addressing this challenge.

## Supporting information

S1 Fig APC of ASIR, ASPR, ASMR and ASDR for global RA from1990 to 2021. (a) ASIR; (b) ASPR; (c) ASMR; (d) ASDR.
(TIFF)

S2 Fig Global comparison of incidence, prevalence, deaths and DALYs counts by age group and their crude rates, 1990 and 2021. (a) Number of incidence and CIR; (b) Number of prevalence and CPR; (c) Number of deaths and CMR; (d) Number of DALYs and CDR.
(TIFF)

S3 Fig Comparison of RA incidence, prevalence, death and DALYs among men and women of different age groups in global in 1990. (a) Incidence;(b)Prevalence;(c) Deaths;(d) DALYs.
(TIFF)

S4 Fig Comparison of RA incidence, prevalence, death and DALYs among men and women of different age groups in global in 2021. (a) Incidence;(b)Prevalence;(c) Deaths;(d) DALYs.
(TIFF)

S5 Fig Comparison of all–age cases with age–standardized incidence, prevalence,deaths, and DALYs in global men and women, 1990–2021. (a) Number of incidence and ASIR; (b) Number of prevalence and ASPR; (c) Number of deaths and ASMR; (d)Number of DALYs and ASMR.
(TIFF)
