# Supplementary figures and images for: Trends in rheumatoid arthritis burden in China and globally, 1990–2021: A longitudinal study based on the GBD database
Source: PLoS One. 2025 May 21;20(5):e0323372. doi: 10.1371/journal.pone.0323372 (PMC12094728; doi:10.1371/journal.pone.0323372)

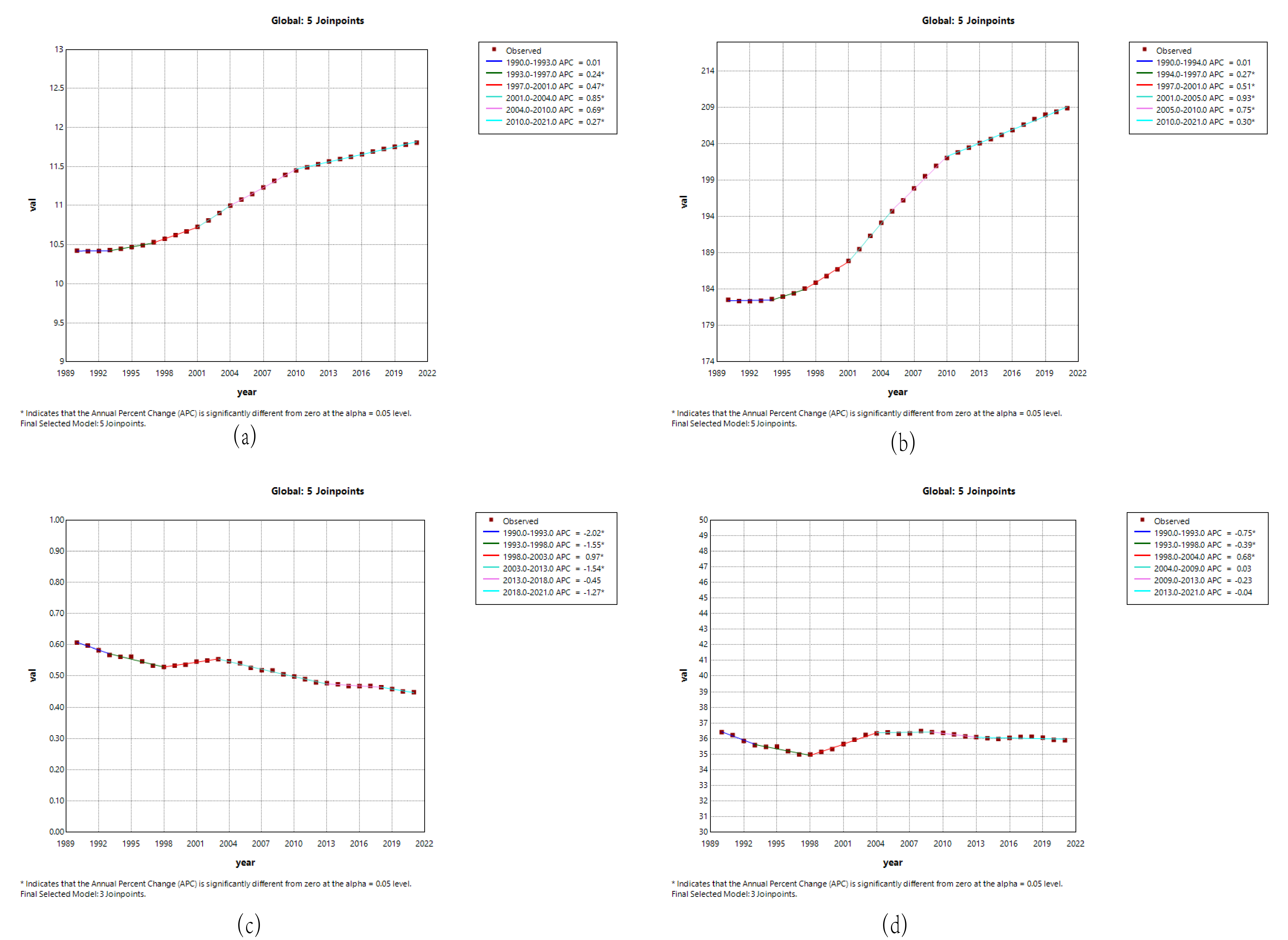

Supplement: S1 Fig — (a) ASIR; (b) ASPR; (c) ASMR; (d) ASDR. (TIFF) [file pone.0323372.s001.tif]

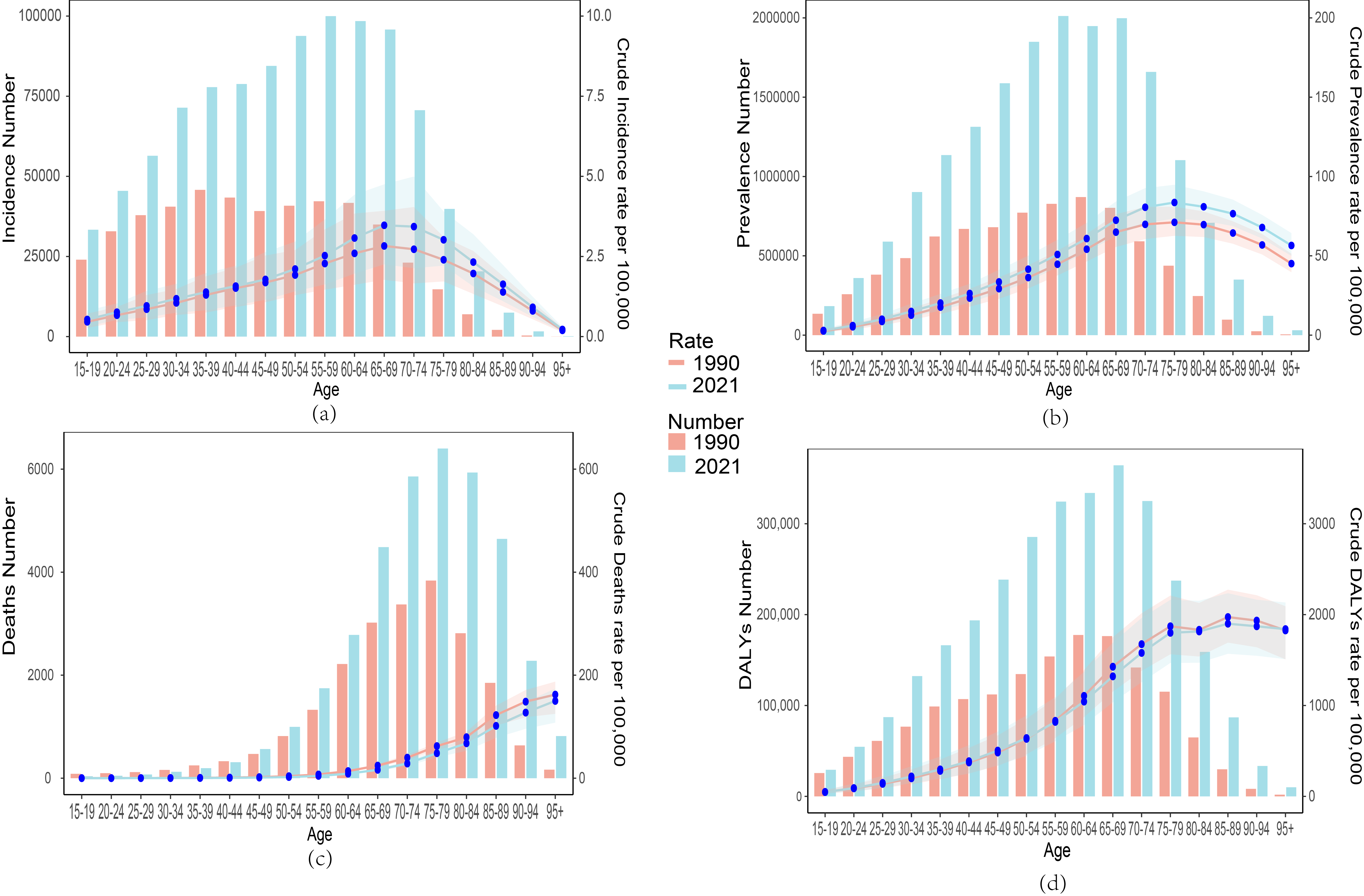

Supplement: S2 Fig — (a) Number of incidence and CIR; (b) Number of prevalence and CPR; (c) Number of deaths and CMR; (d) Number of DALYs and CDR. (TIFF) [file pone.0323372.s002.tif]

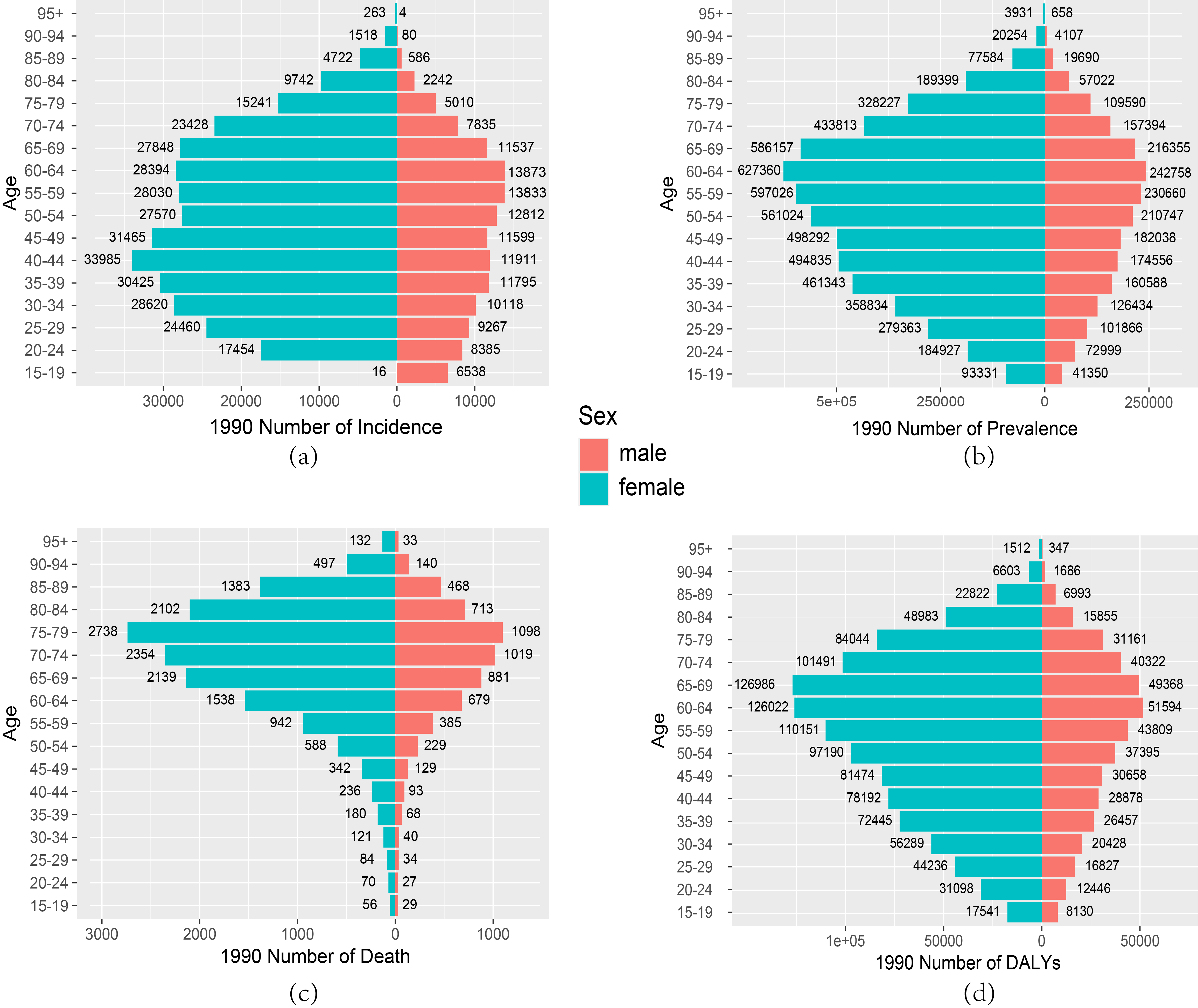

Supplement: S3 Fig — (a) Incidence;(b)Prevalence;(c) Deaths;(d) DALYs. (TIFF) [file pone.0323372.s003.tif]

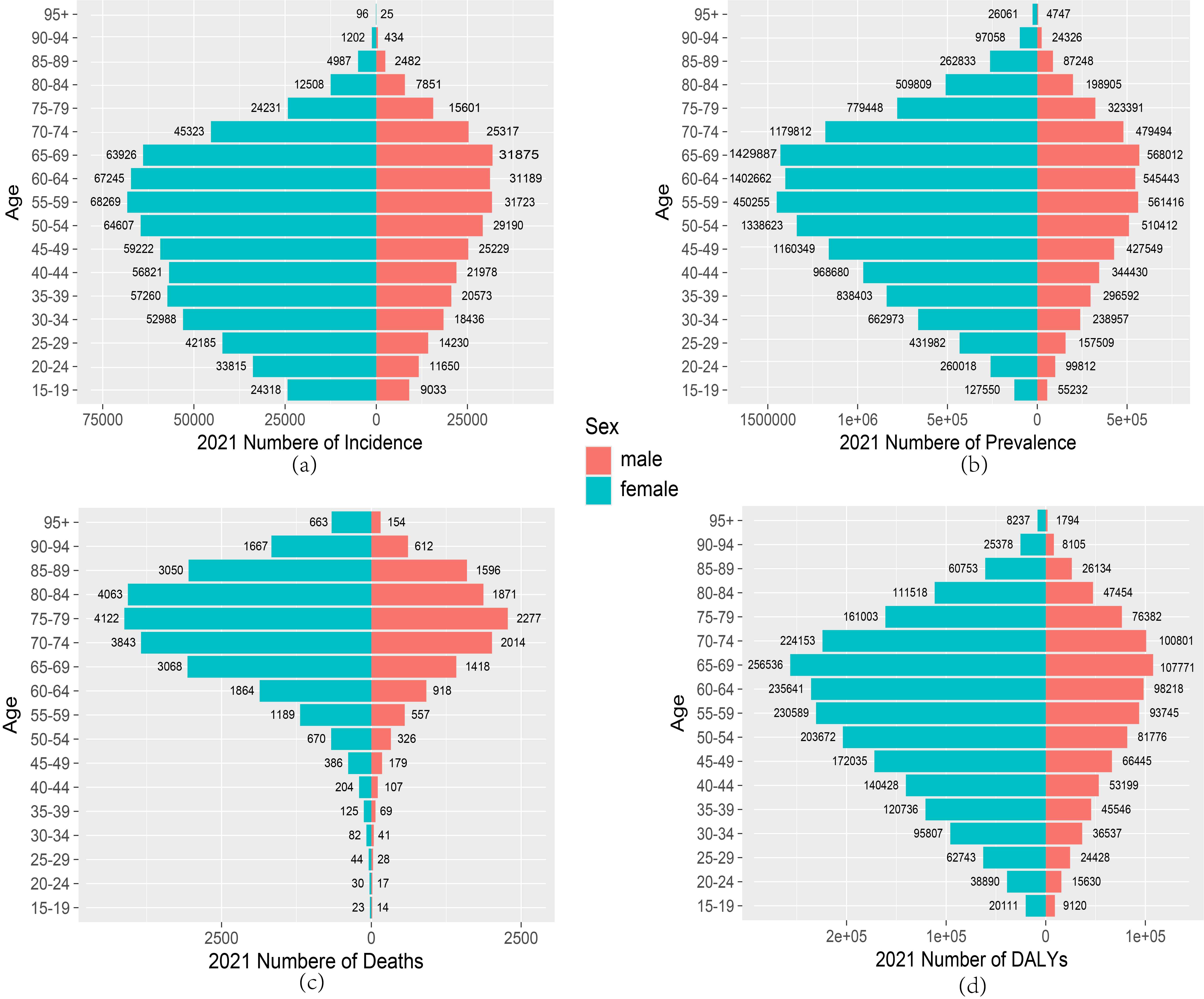

Supplement: S4 Fig — (a) Incidence;(b)Prevalence;(c) Deaths;(d) DALYs. (TIFF) [file pone.0323372.s004.tif]

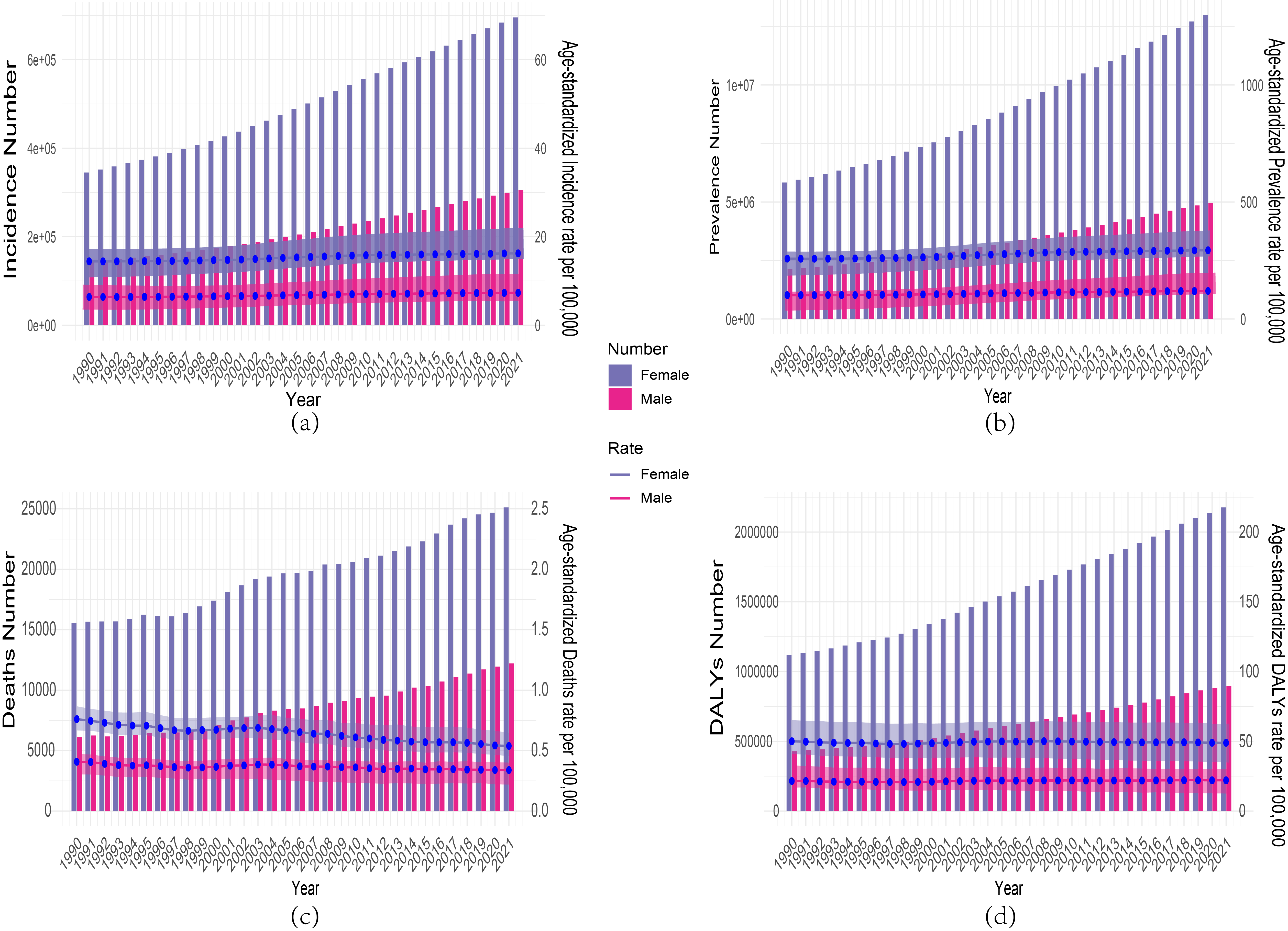

Supplement: S5 Fig — (a) Number of incidence and ASIR; (b) Number of prevalence and ASPR; (c) Number of deaths and ASMR; (d)Number of DALYs and ASMR. (TIFF) [file pone.0323372.s005.tif]
